# Less is more: an ecological and economic point of view on appropriate use of lab testing for COVID patients

**DOI:** 10.4155/bio-2021-0064

**Published:** 2021-08-06

**Authors:** Simona Giulia Signorini, Duilio Brugnoni, Rosella Levaggi, Emirena Garrafa

**Affiliations:** ^1^Department of Laboratory Diagnostics, ASST Spedali Civili, Brescia, Italy; ^2^Department of Economics & Management, University of Brescia, Brescia, Italy; ^3^Department of Clinical & Experimental Sciences, University of Brescia, Brescia, Italy

**Keywords:** appropriateness, biomarkers, cost saving, COVID, environmental cost, hazardous waste, laboratory

The COVID-19 emergency has produced an enormous strain on healthcare systems across the world at different levels [1,2]. This is especially true for hospitals, which are admitting an increasing number of highly infectious patients and facing an unknown illness. Laboratories experienced a sudden change in the demand for tests: the treatment of COVID-19 has required tests that were not frequently requested as routine tests (such as ferritin, D-dimer and other coagulation tests) by hospital physicians, while fear of contagion has reduced demand for other tests (especially for chronic diseases such as diabetes, cancer and autoimmune diseases) because a considerable number of patients did not undergo their usual control. As a consequence, different tests (oncological markers, autoimmunity markers, see [1]) were not requested and reagents expired, creating economic and ecological costs.

The change in the demand for test has required a complete reorganization for big automated labs. A sudden change in the distribution of reagents on the production lines [1,3–5] was in fact necessary to process the tests requierd for COVID-19 patients.

Due to unfamiliarity with the ailment and the fear of missing complications, laboratory tests were used to diagnose and gauge the severity of the patient’s condition because they are easy to perform and provide a quick response without having to move highly infectious patients around (compared with imaging techniques). The laboratory of Spedali Civili in Brescia, as with other laboratories across the world, organized ‘quick pick’ order screens and standard admission templates to facilitate clinicians’ (recruited from different area or specialties) diagnosis and follow-up of COVID-19 patients [1].

Thanks to the enormous literature on this topic, we can now say that for COVID-19, as for other medical fields but exacerbated by the emergency of the pandemic, asking for less, but more appropriate, testing is more. As Baird [6] wrote in his review: “if you ask a stupid question, you get a stupid answer”; we know that the chance of having an abnormal result increases the more tests you take, and the economic and ecological cost of working up a false positive result is always more than asking for fewer, more appropriate tests.

In the same review he referred to ‘wellness testing’. During the COVID-19 outbreak, an example of this would be testing performed for patients with a simple influenza infection or with symptoms that in another time would not be considered meritorious of laboratory diagnostic insight and that potentially represent the origin of a cascade of further investigations.

One year into the crisis, although an effective treatment for COVID-19 is still not available, we have a better understanding of which laboratory tests are appropriate in the diagnosis and the treatment of COVID-19 patients [5,7–9] and which do not add any information on patients’ health status [5,7,8,9]. For example, albumin testing and creatinine clearance were suggested for monitoring COVID-19 patients but are now considered not appropriate to follow the evolution of the disease [5,7,8,9]. Once again, less is more.

Finally, at the onset of the pandemic event, several tests were repeated without respecting the correct interval [10,11] and clinicians performed research without informing the head of the laboratory. This caused an over utilization of tests that could have been better controlled if a closer collaboration were established. More collaboration between different health professionals would have generated less waste of both time and money.

Ensuring that only appropriate laboratory tests are conducted bears several advantages from a medical and an economic point of view. We argue that appropriateness has also an important impact on environmental costs, which is perhaps more relevant than what one might think. In fact, inappropriate tests generate waste of reagents and other lab material that must be disposed of. Most of this waste is hazardous and cannot be recycled so disposal is costly, and usually the only return is heat if it is incinerated using waste-to-energy plants.

In this editorial we used data from the laboratory of Spedali Civili di Brescia (a hospital that, to date, has accepted almost 3000 COVID-19 patients) to analyze the difference in terms of lab exams for treating patients in the emergency period (when literature proposed very few articles about the use of biomarkers, evaluated on small populations, no guidelines were approved and health workers were behaving following their ‘experiences’) compared with what was recently suggested by the literature and the International Federation of Clinical Chemistry (IFCC) guidelines [5,12]. To this purpose, we compared the cost of the routine tests presented in our previous work (which was the appropriate routine for patients hospitalized at the onset of the pandemic) with the cost of the routine test proposed by the IFCC [5] guidelines. This allowed us to determine the saving in the cost for each routine performed. Although, it is nearly impossible to determine the actual cost of each laboratory test in terms of labor, reagents and electricity, it is possible to make an estimate of the extra running costs that could be saved by reducing laboratory tests to only those that are appropriate. For each laboratory test, it is possible to estimate its approximate cost using the reimbursement price list and the additional costs in terms of the hazardous waste that it produces.

Different tests used at the beginning of pandemic event, such as NT-proBNP, calcium, chlorine, cholesterol, electrophoresis, GGT, glucose, IG’s, magnesium, potassium, phosphorus, sodium, birubilin, triglycerides, uric acid, were not recommended anymore. If we compare the reimbursement cost of both routines, we estimate that the cost could be cut by 32%, a percentage close to different examples that abound in the literature [6]. Less testing means more money for the healthcare systems that have suffered worldwide.

We approximated the additional waste with two components – the test tubes and the reagents – by estimating the quantity saved by reducing the number of tests. We weighed tests tubes used in the laboratory and the reagents. We then estimated the weight of the waste produced by the routine suggested in [1] and we compared this with the cost and waste produced by the routine suggested by IFCC, which most of the literature suggests to be appropriate for treating COVID-19 patients. We estimated that waste could be reduced by 20% by implementing the new routine.

A second source of saving derives from the appropriate interval between repeated tests. At the onset of the pandemic, the routine of tests presented in [1] was repeated on each patient about every other day. The new guidelines suggest a leaner routine to be performed every 3 days [4,5]. Since the average length of stay of a COVID patient in our hospital is about 2 weeks [1], the number of appropriate routine batch tests has decreased from 7 to 5.

If we also take into account this reduction, savings increase to 52% for economic costs and to 43% for waste. With the medical information we have at present, we would have been able to reduce lab waste by about 1.473 tons. In fact, in spite of being few hundred grams per each batch of tests, the number of patients for whom these routines have been performed sensibly increases the weight of waste. The carbon footprint of environmental waste depends on the method of disposal and may vary between 21 and 65 kg CO_2_ equivalents per tonne when it is recycled to 1074 kg CO_2_ equivalents per tonne for high temperature incineration [13].

These results are partly due to the reduction in the number of laboratory tests in the appropriate routine and by the fact that tests should be run less frequently; since the average length of stay of COVID-19 patients in hospital is about 2 weeks [14], this makes a substantial difference. Of course, differences can be observed in laboratories that use a different technology compared with the one used in Spedali Civili (Roche Diagnostics, Mannehim, Germany) and different tubes (SarstedAG & Co. KG, Nümbrecht, Germany).

Appropriateness in healthcare is quite important from a medical point of view in order to reduce discomfort to patients and medical errors. To this well-known picture, our analysis adds an important element which will become more important in the years to come. The reduction in inappropriate tests reduces waste in terms of reagents, test tubes full of blood and containers of reagents that need to be discharged as hazardous waste. In the present economic climate where healthcare costs are increasing and income is decreasing, it is essential to avoid waste of financial resources that could be used to treat patients. Even if laboratory costs account only for about 4% of total medical costs, 70% of clinical decisions depends on their results and they; therefore, play a substantial role in driving other nonlaboratory costs [15].

Promoting appropriateness is a complex and neverending issue that is very difficult to achieve. This is particularly true for this emergency where diagnostic uncertainty, fear of legal consequences, insufficient knowledge of laboratory costs and human resource utilization, different levels of healthcare professionals’ training and ‘submerged research’ were all factors in testing. COVID-19, and the need to react to patients’ needs in very uncertain environment, has shown once more the importance of appropriateness. We also think that laboratory professionals should ‘leave the door open’ to other rules by continuing the debate with clinicians to improve appropriate requesting and utilization of laboratory tests as well as the integration of laboratory information with all other diagnostic and clinical data. However, 1 year into this pandemic, we have more information on diagnosis and testing and we should use it to reduce economic and environmental waste because less is more.

## References

[1] Garrafa E, Brugnoni D, Barbaro M Laboratory considerations amidst the COVID-19 outbreak: the Spedali Civili in Brescia experience. Bioanalyis 12(17), 1223–1230 (2020). 10.4155/bio-2020-0109 PMC7315825 32573254

[2] Garrafa E, Levaggi R, Miniaci R, Paolillo C. When fear backfires: emergency department accesses during the Covid-19 pandemic. Health Policy 124(12), 1333–1339 (2020). 33148454 10.1016/j.healthpol.2020.10.006 PMC7584647

[3] Archetti C, Montanelli A, Finazzi D, Caimi L, Garrafa E. Clinical laboratory automation: a case study. J. Public Health Res. 6(1), 31–36 (2017). 10.4081/jphr.2017.881 PMC5477477 28660178

[4] Bohn MK, Lippi G, Horvath A Molecular, serological, and biochemical diagnosis and monitoring of COVID-19: IFCC taskforce evaluation of the latest evidence. Clin. Chem. Lab. Med. 58(7), 1037–1052 (2020). 32459192 10.1515/cclm-2020-0722

[5] Thompson S, Bohn MK, Mancini N IFCC interim guidelines on biochemical/hematological monitoring of COVID-19 patients. Clin. Chem. Lab. Med. 58(12), 2009–2016 (2020). 33027044 10.1515/cclm-2020-1414

[6] Baird G. The laboratory test utilization management toolbox. Biochem. Med. (Zagreb) 24(2), 223–234 (2014). 24969916 10.11613/BM.2014.025 PMC4083574

[7] Lippi G, Plebani M, Henry BM. Thrombocytopenia is associated with severe coronavirus disease 2019 (COVID-19) infections: a meta-analysis. Clin. Chim. Acta 506, 145–148 (2020). 32178975 10.1016/j.cca.2020.03.022 PMC7102663

[8] Heisler M, Choi H, Rosen AB Hospitalizations and deaths among adults with cardiovascular disease who underuse medications because of cost: a longitudinal analysis. Med. Care 48(2), 87–94 (2010). 20068489 10.1097/MLR.0b013e3181c12e53 PMC3034735

[9] Henry BM, De Oliveira MHS, Benoit S, Plebani M, Lippi G. Hematologic, biochemical and immune biomarker abnormalities associated with severe illness and mortality in coronavirus disease 2019 (COVID-19): a meta-analysis. Clin. Chem. Lab. Med. 58(7), 1021–1028 (2020). 32286245 10.1515/cclm-2020-0369

[10] Lang T, Croal B. G17 National minimum retesting intervals in pathology. The Association for Clinical Biochemistry & Laboratory Medicine website (2021). http://www.acb.org.uk

[11] Lang T. Minimum retesting intervals in practice: 10 years experience. Clin. Chem. Lab. Med. 59(1), 39–50 (2021). 10.1515/cclm-2020-0660 32892171

[12] Lei R, Mohan C. Immunological biomarkers of COVID-19. Crit. Rev. Immunol. 40(6), 497–512 (2020). 33900694 10.1615/CritRevImmunol.2020035652

[13] Rizan C, Bhutta MF, Reed M, Lillywhite R. The carbon footprint of waste streams in a UK hospital. J. Clean. Prod. 286, 125446 (2021).

[14] Garrafa E, Vezzoli M, Ravanelli M Early prediction of in-hospital death of COVID-19 patients: a machine-learning model based 1 on age, blood analyses, and chest x-ray score 2. medRxiv, (2021) (Epub ahead of print). 10.7554/eLife.70640 PMC8550757 34661530

[15] Hallworth MJ, Epner PL, Ebert C Current evidence and future perspectives on the effective practice of patient-centered laboratory medicine. Clin. Chem. 61(4), 589–599 (2015). 25646214 10.1373/clinchem.2014.232629

